# A multi‐layered nerve guidance conduit design adapted to facilitate surgical implantation

**DOI:** 10.1002/hsr2.86

**Published:** 2018-10-31

**Authors:** Kayla Belanger, Guy Schlatter, Anne Hébraud, Frédéric Marin, Sylvie Testelin, Stéphanie Dakpé, Bernard Devauchelle, Christophe Egles

**Affiliations:** ^1^ UMR 7338, Biomécanique et Bioingénierie, Centre de recherches de Royallieu Sorbonne Universités, Université de Technologie de Compiègne, CNRS Compiègne cedex France; ^2^ ICPEES Institut de Chimie et Procédés pour l'Energie, l'Environnement et la Santé, UMR 7515, CNRS Université de Strasbourg Strasbourg cedex France; ^3^ Facing Faces Institute, Amiens University Hospital Center Amiens Cedex 1 France; ^4^ Tufts University, School of Dental Medicine Boston MA USA

**Keywords:** electrospinning, implant, nerve guidance conduit, nerve regeneration, silk fibroin

## Abstract

**Background and Aims:**

The gold standard procedure after a severe nerve injury is the nerve autograft, yet this technique has drawbacks. In recent years, progress has been made in the development of artificial nerve guides to replace the autograft, but no device has been able to demonstrate superiority. The present study introduces an adaptable foundation design for peripheral nerve regeneration.

**Methods:**

Silk fibroin was electrospun, creating a tri‐layered material with aligned fiber surfaces and a randomly deposited fiber interior. This material was rolled into a micro‐channeled conduit, which was then enveloped by a jacket layer of the same tri‐layered material.

**Results:**

The proposed implant design succeeds in incorporating various desirable aspects of synthetic nerve guides, while facilitating the surgical implantation process for medical application. The aligned fiber surfaces of the conduit support axon guidance, while the tri‐layered architecture improves its structural integrity compared with a fully aligned fiber material. Moreover, the jacket layer creates a small niche on each end which facilitates surgical implantation. An in vivo study in rats showed that nerve regeneration using this device was comparable to results after direct suture.

**Conclusion:**

This proof‐of‐principle study, therefore, advances the development of tissue engineered nerve grafts by creating an optimized guidance conduit design capable of successful nerve regeneration.

## 1 INTRODUCTION

The nervous system is made up of the central nervous system (CNS) and the peripheral nervous system (PNS). The CNS includes the brain and spinal cord, while the PNS includes all nerves that branch out from the CNS. Nerves in the PNS contain sensory, or afferent, neurons that carry information to the CNS, and motor, or efferent, neurons that transmit information from the CNS to the periphery. The cell bodies of motor and sensory neurons are located in the spinal cord and in dorsal root ganglia, respectively, and each has long extensions called axons that innervate the various organs throughout the body. Due to environmental constraints in the CNS, spontaneous neuronal repair after injury does not occur.^1^, ^2^ The PNS, on the other hand, differs in this characteristic, supporting axon regeneration after a minor injury to the nerve, but if the injury is severe, spontaneous regeneration will not be successful.

After a severe injury to a peripheral nerve, the axon segments distal to the trauma site begin to degrade, which results in a local loss of function as organs are denervated, during a process called Wallerian degeneration.^3^, ^4^ Once the distal axon segments degrade, the proximal axon stubs begin to regrow towards their respective innervation targets, assisted by biochemical cues and structural guidance from proliferating Schwann cells.^3^, ^5^ In many minor nerve injury cases, nerves will regenerate naturally, resulting in functional recovery without the need for surgical intervention. However, for more serious injuries, where the gap between uninjured nerve segments cannot achieve a tension‐free coaptation, a nerve graft must be implanted in order to bridge the gap between the proximal and distal segments of the nerve.^6^

In North America, between roughly 50 000 and 200 000 surgeries for nerve repair are performed each year.^7^ The current gold standard for nerve repair when direct end‐to‐end suture is impossible, is the nerve autograft, which consists of grafting the damaged nerve with a nerve sample taken directly from the patient; samples are most often taken from the sural nerve located in the leg and the medial antebrachial cutaneous nerve located in the arm.^8^ Other sensory nerve harvesting locations may be considered by surgeons, such as the superficial cervical plexus.^9^ Not only is this method's effectiveness in nerve regeneration limited, resulting in full functional recovery in only half of cases, but it also has several other drawbacks, such as the need for multiple operations, limited donor tissue availability, loss of function at the donor site, and the risk of developing a painful neuroma at the donor site.^5^, ^10^ Before following through with a nerve autograft procedure, it is important that the surgeon informs the patient of the consequential postoperative sensory loss in either the lateral portion of the foot or the medial portion of the mid‐forearm (a common side effect of an autograft from the sural nerve and medial antebrachial cutaneous nerve, respectively), as the loss of sensory function in one area may be more or less significant for each patient.

In order to overcome the drawbacks presented by the autograft, researchers have turned to tissue engineering in order to develop a more effective alternative, using biomaterials.^11^ In particular, the natural polymer silk fibroin is a promising material for the fabrication of a nerve guidance conduit, since it is biocompatible, biodegradable, easily functionalized, easily chemically modified, and has robust mechanical properties compared with other natural materials.^12^ In addition to the choice of biomaterials, an effective nerve guidance conduit must provide an environment that encourages healthy, guided axon regrowth.^5^ The conduit must, therefore, succeed in preventing the regrowing axons from straying far from their original paths towards their innervation targets. If regenerated neurons were misdirected during regrowth, not only will functional recovery be significantly reduced in intensity, but the patient may also require sensory and motor re‐education therapy in order for the brain to recognize an entirely new input, as neurons that once innervated one area of the body may have reinnervated a different area.^8^ In cases of minor injuries to the nerve, axons are directed by the naturally realigning Schwann cells that release biological cues, thus attracting growth cones and successfully leading the axons along their original paths towards their innervation targets.^13^ The autograft has an advantage regarding guidance capabilities due to the numerous micro‐channels in the predeveloped fascicles which increase the surface area to volume ratio and lower the possibility of axons straying from a predetermined route.^14^, ^15^ Most artificial nerve guidance conduits on the market, however, are hollow tubes that do not provide as much mechanical support.^14^

The epineurium is the outermost layer of the nerve and is made up of dense connective tissue which houses the nerve fascicle; these have their own layer of protective connective tissue called the perineurium. In most cases, autografts or nerve guidance conduits are sutured at the epineurium of the nerve at the proximal and distal nerve stumps in order to connect a severed nerve.^15^ In some cases, several nerve grafts are sutured directly at the perineurium of each fascicle with the aim of better fascicle matching. Micro‐suture at the perineurium, however, results in increased trauma and scarring to the nerve, and there is no consistent evidence of superior results to epineurial sutures.^6^, ^15^ However, after a nerve is severed, surgeons observe that the epineurium will naturally retract a small percentage further than the nerve fascicles due to a release in tension. This consequence poses a challenge for surgeons to correctly position implants with blunt edges, such as an autologous nerve graft. This is because as the epineurium is stretched to connect with the outer layer of a blunt‐edged implant, the fascicles may become deformed, weakening the prospect of successful neuron guidance. Hollow nerve guidance conduits, such as Neuroflex or NeuraGen, overcome this obstacle, since the protruding nerve fascicles are simply inserted into the hollow cavity of the implant.^16^ However, hollow conduits provide minimal directionality due to lower physical support.

Finally, the mechanical strength of the device is an important parameter that must be considered.^17^ The implant must be resistant to all forces acting upon the implant during and after implantation. For example, a weak resistance to tensile forces after surgery can lead to poor regeneration, and surgeons request that regenerating neurons travel a longer distance through a longer implant, as opposed to a shorter implant that is put under excessive stress.^8^, ^15^ Therefore, the implant must uphold a certain stability while under tensile stress in order to achieve a conduit with a relatively small length and an environment that will promote healthy neuron growth. The material must also withstand the trauma of suture during the surgery and, therefore, must possess strong tear strength in order to facilitate successful implantation.

Silk fibroin extracted from the *Bombys mori* silk worm cocoons was chosen for this study because of its numerous advantageous properties as a natural material. Once the fibroin protein is purified from the raw silk cocoons, it is a biocompatible material that generates a weaker inflammatory response than that of both collagen and PLA, which are commonly investigated biomaterials for nerve guidance conduit fabrication.^5^, ^12^ Silk fibroin is an interesting biomaterial for this study also because it is easily chemically modifiable as well as functionalizable with diverse substances^18^; material functionalization could ultimately be optimized to yield a superior biomaterial complex. In addition, the degradation properties of silk fibroin can be controlled during material fabrication. Hu et al demonstrated that increasing the amount of β‐sheets in the protein secondary structure ultimately slows biodegradation.^19^ Finally, silk fibroin has already been FDA approved as a biological suture material.

The design of the device presented in this study takes several factors into consideration. First, the material and material structure were chosen for biocompatibility, versatility, and mechanical integrity. Silk fibroin was electrospun to create a complex, tri‐layered nanofiber material optimizing parameters to allow both surface alignment and good mechanical strength. Second, micro‐channels were included in the fabrication of the nerve guidance conduit in order to incorporate a significant advantage valued in the nerve autograft. Finally, a jacket layer was added to the multi‐channeled conduit in order to incorporate the principal advantage to hollow nerve guides, which is to facilitate the surgical procedure by allowing a more straightforward epineurial micro‐suture technique. Therefore, the goal of this study was to develop an adaptable implant foundation design capable of providing enhanced guidance to regenerating neurons that also caters to the needs of the surgeon during implantation.

## 2 METHODS

### 2.1 Preparation of silk fibroin solution

A 10 wt% silk fibroin solution was obtained using a previously established protocol.^33^ Briefly, silk cocoons from the Bombyx mori silkworm were cut into small pieces and boiled for 30 minutes in a 0.02 M Na_2_CO_3_ aqueous solution. The silk fibroin fibers were rinsed three times in DI water and then allowed to dry at room temperature. The dry fibroin fibers were dissolved in a 9.3 M solution of LiBr for up to 4 hours at 60°C. The fibroin/LiBr solution was dialyzed against DI water using Slide‐a‐Lyzer dialysis cassettes (3500 MWCO, Thermo Fisher Scientific, 87725) for 3 days, to remove salts. The fibroin solution was centrifuged twice to remove solid contaminants. The final concentration was about 6 wt%. The solution was concentrated to 10 wt% by evaporation of the solvent. The final solution was stored at 4°C for up to 6 weeks.

### 2.2 Electrospinning

A 10 wt% SF solution was mixed at a volume ratio of 4:1 with a 5 wt% solution of poly (ethylene oxide) (PEO, average M_v_ ~900 000, Sigma‐Aldrich, 25322‐68‐3) resulting in a 8:1 wt:wt SF:PEO spinning solution. The spinning solution was dispensed through a 19 G stainless steel spinneret (Ramé‐Hart Instrument Co.) at a flow rate of 1 mL·h^−1^, while a voltage between 10 and 15 kV was administered. An electrospinning rotating drum collector made in‐house, used to collect the silk fibers, had a diameter of 12.8 cm and a width of 3 cm. The drum was positioned on the same axis as the spinneret at a distance of 12.5 cm from the tip of the spinneret. The entire system was enclosed in a Plexiglas containment area with a controlled humidity range of 25% to 35%. To obtain an aligned fiber material, the spinning solution was dispensed continuously for 90 minutes while the collector rotated at 4000 RPM, resulting in a surface velocity of 26.8 m·s^−1^. To obtain a tri‐layered material consisting of a first layer of aligned fibers, a layer of randomly deposited fibers, and a final layer of aligned fibers, the spinning solution was dispensed for a total of 90 minutes: 30 minutes with a collector rotation speed of 4000 RPM, followed immediately by 30 minutes at 400 RPM (surface velocity of 2.68 m·s^−1^), then followed by 30 minutes at 4000 RPM. To obtain a randomly deposited fiber material, the spinning solution was dispensed continuously for 90 minutes with a collector rotation speed of 400 RPM. Aluminum foil was used to cover the collector surface before each process to allow for easy sample recovery.

### 2.3 Implant fabrication—jacketed, multi‐channel design

The implant fabrication process is shown in Figure S1. The tri‐layered electrospun material was carefully peeled from the collector, and a 5 mm (parallel to aligned fibers) by 3 cm (perpendicular to aligned fibers) rectangle of the material was cut using a scalpel blade. The material was then rolled while adding a Teflon‐coated stick (0.2‐mm diameter) after every full rotation. Once completely rolled, the tube was immediately immersed in methanol for 5 minutes to induce β‐sheet formation. The tube was allowed to air‐dry for 1 hour, and the Teflon‐coated sticks were then removed, resulting in a 5‐mm‐long multi‐channeled tube.

From the tri‐layered electrospun material, a 7 mm (parallel to aligned fibers) by 3 cm (perpendicular to aligned fibers) rectangle was cut. The multi‐channel tube was placed at the bottom‐center of this rectangle (aligned fibers in the same direction). The larger material was then rolled around the tube for 3 rotations to create a “jacket.” The edge was tightly pressed to the tube, and the entire device was water vapor annealed at room temperature for 4 hours to induce β‐sheet formation. The implants were immersed in Milli‐Q water overnight at 37°C and then rinsed three times in order to eliminate traces of PEO. The implants were then sterilized in 70% ethanol overnight, rinsed three times with sterile water, and immersed in sterile PBS for storage.

### 2.4 Scanning electron microscope imaging

Electrospun fiber materials were sputter coated with gold (Q150RS, Quorum technologies) before SEM observation. The surface and the edge of the fibrous materials were then observed by SEM (TECSAN Vega 3 LMU) using the Everhart‐Thornley detector under high vacuum mode using an accelerating voltage of 5 kV and working distances in the range of 6 to 8 mm. To observe the three layers of the tri‐layered material samples, the material was frozen in liquid nitrogen before being sectioned for a clean, blunt edge for analyses.

### 2.5 Mechanical strength testing

For tensile strength tests, purely aligned, randomly deposited, and tri‐layered electrospun material samples were rolled four rotations to produce a hollow tube. The tubes were water vapor annealed for 4 hours at room temperature and then immersed in Milli‐Q water overnight at 37°C to extract the PEO from the fibers. The tubes were subsequently rinsed and then immersed in PBS prior to testing. Hydrated material samples were consecutively secured lengthwise between the upper and lower holding grips with a gauge length of 3 mm. Each trial was carried out at a cross‐head speed of 0.06 mm·s^−1^ while recording load measurements every 100 ms until rupture. Assays for each material were done in triplicate. All values are represented by mean ± standard deviation.

In order to test the tear strength of the materials, aligned and tri‐layered material samples wear punctured with a 9‐0 round bodied suture needle with a polyamide 6/6 thread 1 mm from the materials' edge. The suture thread was trimmed on both sides of the puncture, but not knotted. The threaded material was secured in the lower holding grips at the opposite edge of the puncture, while the two free suture threads were secured in the upper holding grips. The sutured material was adjusted automatically in order to assure equal tension between both sides of the suture thread. The tensile force of the system was then measured with a cross‐head speed of 0.06 mm·s^−1^ up to a maximum displacement of 4 mm. Assays were done in triplicate. All values are represented by mean ± standard deviation.

### 2.6 Fiber diameter and angle analysis

From SEM images at randomly chosen areas of the aligned material surface, 100 fiber diameters were measured, and 50 fiber angles were measured using ImageJ image analysis software (version 1.50i). Diameters of each fiber were measured at the center of the SEM image unless otherwise hidden from view at the image's center. Fiber angles were measured by drawing straight lines along the fiber and the y axis of the image. All angles are expressed relative to the primary alignment of the aligned fibers (0°), which was calculated by subtracting the average fiber angle from the measured fiber angle relative to the y axis of the image. All calculated fiber angles were from a single SEM image. All values are represented by mean ± standard deviation.

### 2.7 In vitro study

Electrospun silk fibroin fibers and glass coverslips were immersed in 70% ethanol for 24 hours and allowed to dry prior to cell seeding. Rat Schwann cells (Innoprot, P10301) were added to the surface of electrospun silk fibroin fibers and to the surface of glass coverslips at a density of 10 000 cells/cm^2^. Once cells had adhered to the surfaces, culture media supplied by Innoprot (P60123‐4) for rat Schwann cell cultures and supplemented with penicillin (10 000 IU/mL) and streptomycin (10 000 μg/mL) was added to each sample. Culture plates were incubated at 37°C with a CO_2_ content of 5% for 48 hours. Experiments were done in triplicate.

Rat Schwann cells provided by Innoprot are isolated from postnatal day 8 CD® IGS rat sciatic nerve at ScienCell Research Laboratories and cryopreserved as primary cultures. Vials are delivered frozen, and cells were used within 3 months of delivery.

### 2.8 Surgery

Twelve 6‐week‐old, male Sprague Dawley rats were each anesthetized with Vetflurane (4.5% during induction and 3.5% during surgery, Virbac) throughout the entirety of the operation. The rats' right hind limbs were each shaved and sterilized. For each rat, an incision parallel to the femur was made, and the sciatic nerve was exposed, isolated, and fixed with two micro clips 10 mm apart. The nerve was severed with surgical scissors at two points 5 mm apart between the micro clips, and the extracted portion of the nerve was discarded.

For the implantation of nerve guidance conduits made from aligned fiber materials without jacket layers (four animals), the edge of the implant was sutured (Ethicon Ethilon Polyamide 6/6 suture, 9‐0 round bodied) at four points to the distal segment's epineurium (at 0°, 90°, 180°, and 270°). The proximal nerve segment was then lined up with implant's proximal end. The edge of the implant was subsequently sutured at four points to the proximal segment's epineurium (at 0°, 90°, 180°, and 270°).

For the implantation of nerve guidance conduits made from the tri‐layered fiber material with a jacket layer (four animals), the implant outer layer was sutured (Ethicon Ethilon Polyamide 6/6 suture, 9‐0 round bodied) twice to the distal segment's epineurium (at 0° and 180°) enveloping the epineurium and nerves fascicles. The exposed epineurium and fascicles of the proximal nerve segment's severed edge were inserted into the small cavity at the proximal end of the implant, and the implant outer layer was sutured twice to the epineurium (at 0° and 180°). The operated area was then cleaned, and the wound was closed and sutured.

For direct sutures (four animals), distal and proximal nerve segments were lined up and sutured (Ethicon Ethilon Polyamide 6/6 suture, 9‐0 round bodied) end‐to‐end at four points (at 0°, 90°, 180°, and 270°) at each segment's epineurium.

After suture, the operated areas were cleaned, and the wound was closed and sutured.

### 2.9 In vivo study

Procedures and animal treatment complied with the principles and guidelines of the French legislation on animal welfare (n° 2013‐118) and gained approval (n°96) by the Ethics Committee “Comité Régional d'Ethique en Matière d'Expérimentation Animale de Picardie (CREMEAP)”. Sprague Dawley rats (6 weeks) were purchased from Janvier Labs (Saint‐Berthevin, France). Rats were housed in ventilate cages NexGen (Allentown, France) in a temperature‐controlled room (21 ± 1°C) with a 12‐hour light/12‐hour Darkness cycle. Food and water were available ad libitum.

Two experimental groups plus a control group were studied in an 8‐month in vivo study on nerve regeneration of the sciatic nerve in the Sprague Dawley rat. Experimental group 1 included four animals with a jacketed silk fibroin‐based nerve guidance conduit implanted between two severed sciatic nerve segments, as previously described in the Surgery section. Two animals were analyzed at month 4 of the study, and two animals were analyzed at month 8 of the study.

Experimental group 2 included four animals whose sciatic nerve in the right hind limb was severed as previously described in the Surgery section and subsequently secured end‐to‐end with four micro‐sutures at 0°, 90°, 180°, and 270°. Two animals were analyzed at month 4 of the study, and two animals were analyzed at month 8 of the study. The operated area was then cleaned, and the wound was closed and sutured.

The control group animal was not operated on.

Half of the animals from each experimental group were euthanized at 4 months after surgery and half at 8 month after surgery. Segments of the sciatic nerve cut within 10 mm distal and proximal to the site of injury were harvested from each animal prior to euthanization and immersed in 4% formaldehyde for at least 3 days.

### 2.10 Tissue sectioning

Nerve segments were immersed in subsequent baths of 15% sucrose solution and 30% sucrose solution then flash frozen in OCT compound (Fisher Healthcare, 23‐730‐571). Nerve sections with a thickness of 8 μm were collected using a cryomicrotome (Leica CM 3050 S).

### 2.11 Immunostaining and imaging

Schwann cells were fixed in 4% formaldehyde and immunostained using DAPI (Sigma Aldrich, 28718‐90‐3) and Alexa Fluor 488 phalloidin (Thermo Fisher Scientific, A12379) for visualization of nucleic acids and actin filaments, respectively. Samples were visualized with an epifluorescence microscope (Leica DMI 6000B, 20x/0.40).

Tissue sections were immunostained either with a combination of DAPI, anti‐β‐tubulin III (Sigma Aldrich, T2200), and Alexa Fluor 488 phalloidin for visualization of nucleic acids, β‐tubulin III, and actin filaments respectively, or a combination of DAPI, anti‐β‐tubulin III, and anti‐myelin protein zero (Abcam, Ab134439) for visualization of nucleic acids, β‐tubulin III, and protein zero, respectively. Immunostaining procedures were carried out according to the manufacturers' instructions.

Samples were visualized with either an epifluorescence microscope (Leica DMI 6000B, 20x/0.40) or a confocal microscope (Zeiss LSM 710, 63x/1.40 Oil DIC) using z‐stacking method. Fifty nerve fiber diameters and 50 myelin sheath thicknesses in each sample were measured using ImageJ image analysis software (version 1.50i). All values are represented by mean ± standard deviation.

## 3 RESULTS

### 3.1 Nerve guidance conduit design

**Figure 1:**
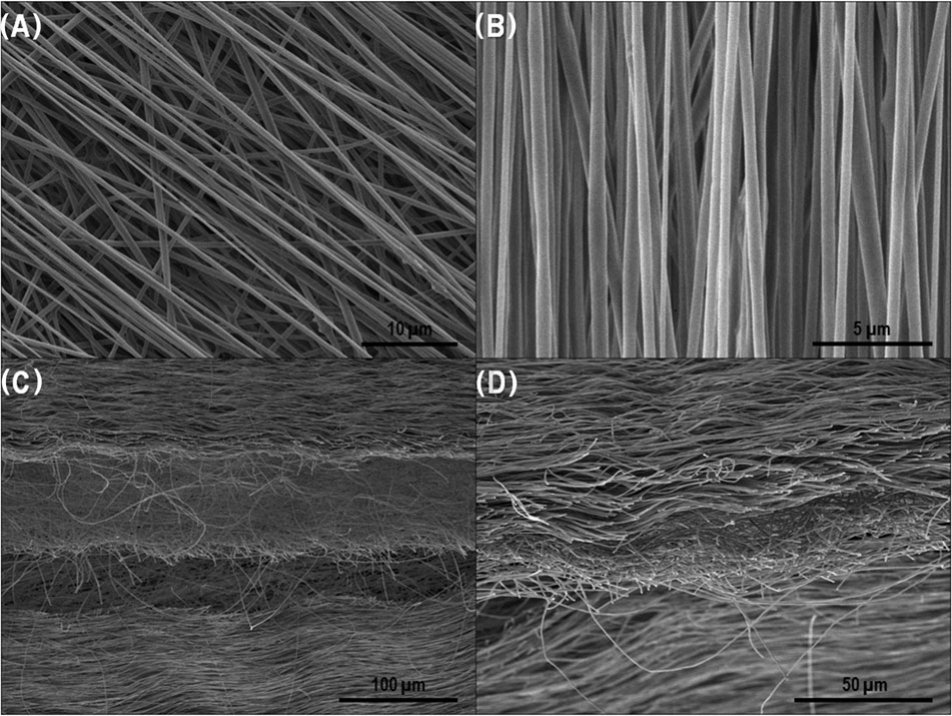
SEM images of A, beginning of the final aligned layer deposited on center unaligned layer; B, material's aligned surface layer; C and D, cross sections of the tri‐layered material after being snap frozen in liquid nitrogen and sectioned longitudinally

After a total of 3 × 30 minutes of electrospinning, a tri‐layered fibrous silk fibroin material 3 cm wide and 80 μm (±10 μm) thick was obtained. The tri‐layered material consisted of an internal layer of randomly deposited fibers, which was sandwiched between two layers of aligned fibers. Fiber diameters were found to be 417 ± 134 nm, with 94% of diameters within the range of 200 nm and 600 nm. In the aligned fiber layers, 86% of fiber angles were found to be within ±5° of the primary alignment. The tri‐layered material exhibited three distinct layers visible through SEM analyses (Figure 1). The aligned‐fiber surfaces of the tri‐layered material were also shown to completely cover the random fiber center layer (Figure 1B), assuring that the guidance factor of this material was not compromised.

**Figure 2:**
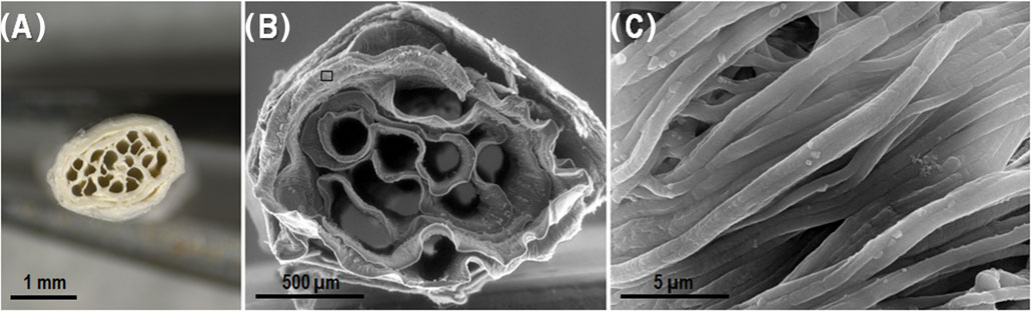
A, Cross section of implant; B, SEM cross section of implant; and C, close‐up of material surface from square frame in image B

A multi‐channeled conduit capable of maintaining its structure after water vapor annealing (necessary to induce the formation of silk fibroin β‐sheets) was obtained and is shown in Figure 2.

### 3.2 Mechanical tests

**Figure 3:**
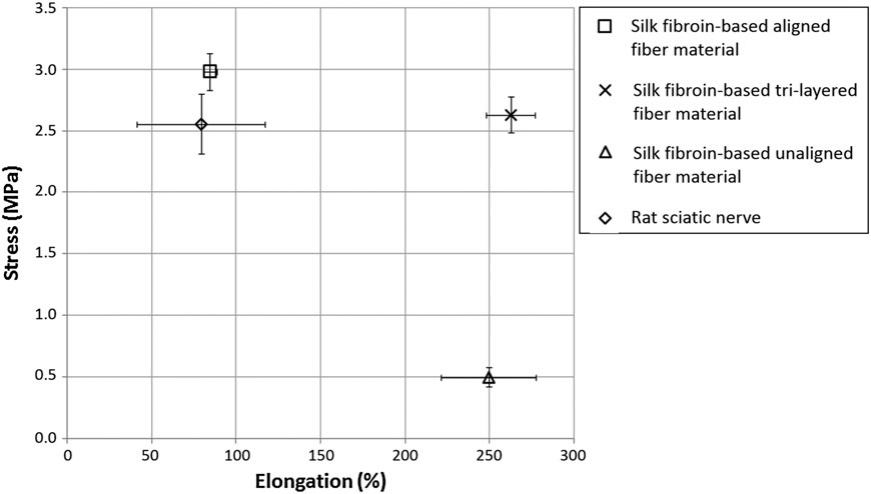
Average stress‐elongation measurements at maximum stress values of silk materials and rat sciatic nerve. (Error bars represent standard deviation of triplicate tests)

**Figure 4:**
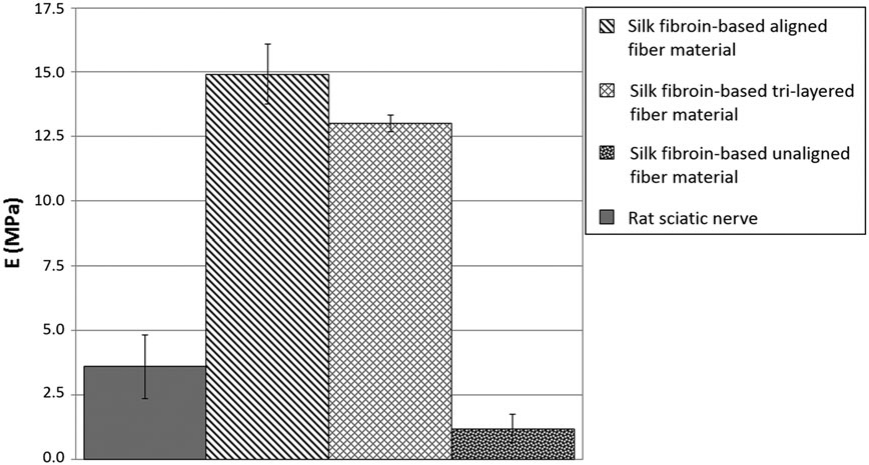
Average Young's modulus measurements of silk materials and rat sciatic nerve. (Error bars represent standard deviation of triplicate tests)

**Figure 5:**
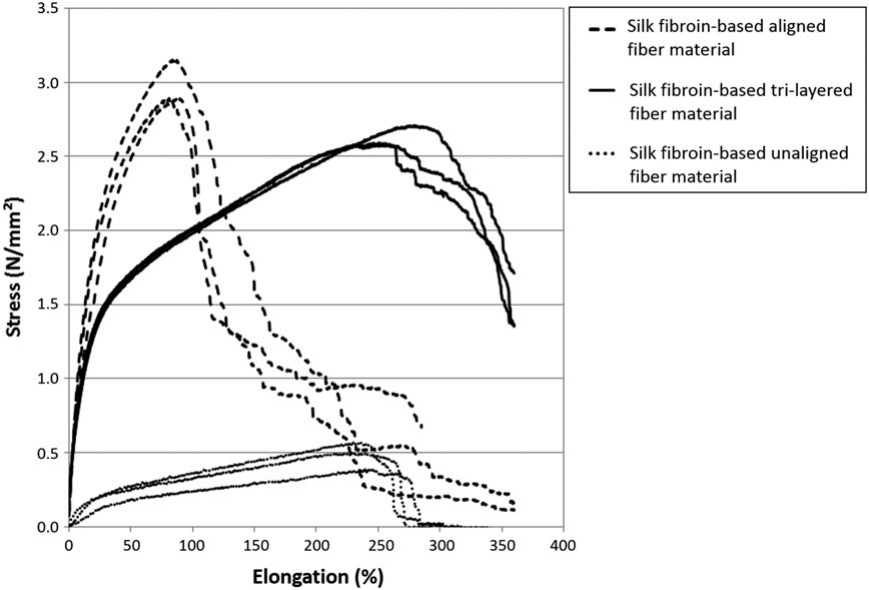
Stress‐Elongation curves of aligned, tri‐layered, and unaligned material samples. Results from triplicate tests are plotted.

Ultimate tensile strength and Young's modulus characterizations of aligned, random, and tri‐layered material samples are represented in Figures 3, 4, and 5, respectively. For the samples with aligned fibers, the tensile strength tests were carried out according to the direction of the fibers. The considered apparent tensile stress σ is calculated from the cross‐sectional area (A = 0.08 mm × 30 mm) of the sample.

The aligned and tri‐layered material samples were shown to possess similar average ultimate strength measurements of 2.9 and 2.6 MPa, respectively, compared with that of the rat sciatic nerve (2.6 MPa) and significantly higher than that of the randomly deposited material (ie, unaligned), demonstrating an average ultimate strength of 0.5 MPa (Figure 3). The aligned material was found to be significantly more resistant to elongation than both the randomly deposited and tri‐layered material samples, exhibiting an average elongation of 85% at the ultimate strength. This behavior is comparable to the rat sciatic nerve, which had an elongation of 80% at ultimate strength. Randomly deposited and tri‐layered material samples had similar elongation values at ultimate strength (250% and 263%, respectively), which were, thus, significantly higher than that of aligned fibers. This behavior can be explained by the rearrangement and the alignment of the random fibers with respect to the tensile direction (Figure 3).^20^ Young's modulus values of the aligned, tri‐layered, and randomly deposited materials (shown in Figure 4) were found to be 14.9, 13.0, and 1.2 MPa, respectively, compared with the Young's modulus of the rat sciatic nerve at 3.6 MPa. A higher Young's modulus found in both the aligned and tri‐layered fiber materials indicates a higher resistance to elastic deformation compared with the lower measurements of both the randomly deposited material and the sciatic nerve sample. Finally, in comparison to randomly deposited and tri‐layered material samples, aligned material samples were found to be more brittle, demonstrated by complete rupture very shortly after reaching the maximum stress (Figure 5). The tri‐layered material samples preserved continuity long after initial rupture (Figure 5).

**Figure 6:**
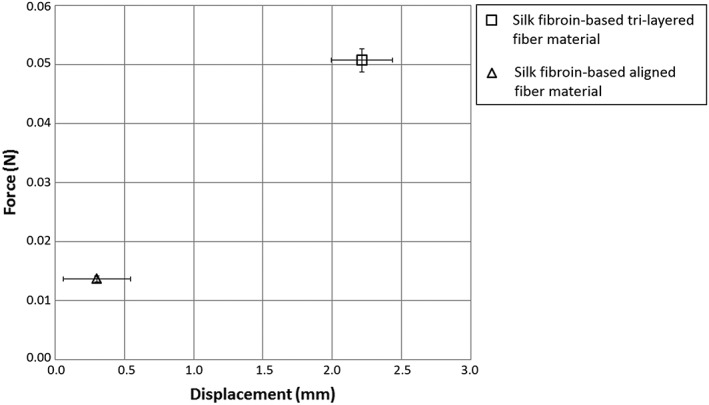
Average displacement and tensile force measurements at the average maximum force withstood by the sutured aligned and tri‐layered silk materials. (Error bars represent standard deviation of triplicate tests)

Tear strength characterizations of both aligned and tri‐layered materials are shown in Figure 6. The sutured tri‐layered material was able to resist an average maximum force of 50.7 mN ± 2.0 mN, which was reached at a displacement of 2.2 mm ± 0.2 mm. The aligned material was able to withstand a maximum force of 13.7 mN ± 0.6 mN, which was reached at a displacement of 0.3 mm ± 0.2 mm.

### 3.3 In vitro study

**Figure 7:**
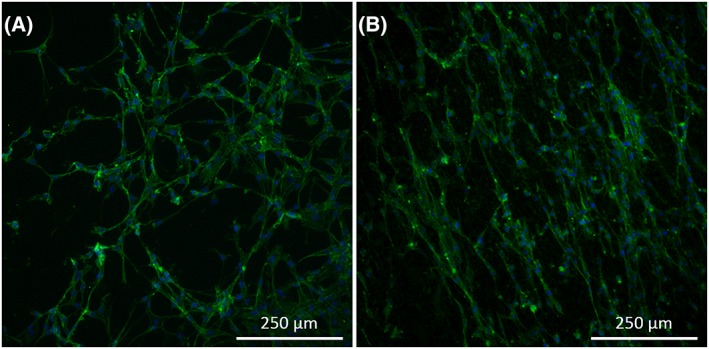
Rat Schwann cells after 48 h in culture on A, glass coverslip and B, electrospun aligned silk fibroin fibers, visualized with fluorescence microscopy at 10x magnification. Nuclei are represented in blue (DAPI) and cell cytoskeleton is represented in green (Alexa Fluor™ 488 phalloidin). Images representative of triplicate tests performed independently.

After Wallerian degeneration, Schwann cells must be able to migrate through the nerve guidance conduit in order to create paths towards which the regeneration nerve fibers will be guided. Therefore, rat Schwann cells were seeded on the aligned silk fibroin fiber material surface in order to analyze cell behavior on this material in vitro. Compared with control samples consisting of rat Schwann cells seeded on glass cover slips, Schwann cells appeared to thrive similarly on each sample surface yet exhibiting morphological differences (see Figure 7). Schwann cells on glass coverslip surfaces exhibited cytoskeletal extensions interacting with neighboring cells in no apparent organization, creating a randomized network of Schwann cells. In contrast, Schwann cells seeded on aligned fibroin fiber surfaces aligned themselves on the fibers. These cells stretched along the fiber and interacted primarily with cells along the same fiber length creating strings of cells after only 48 hours in culture.

### 3.4 Surgery

**Figure 8:**
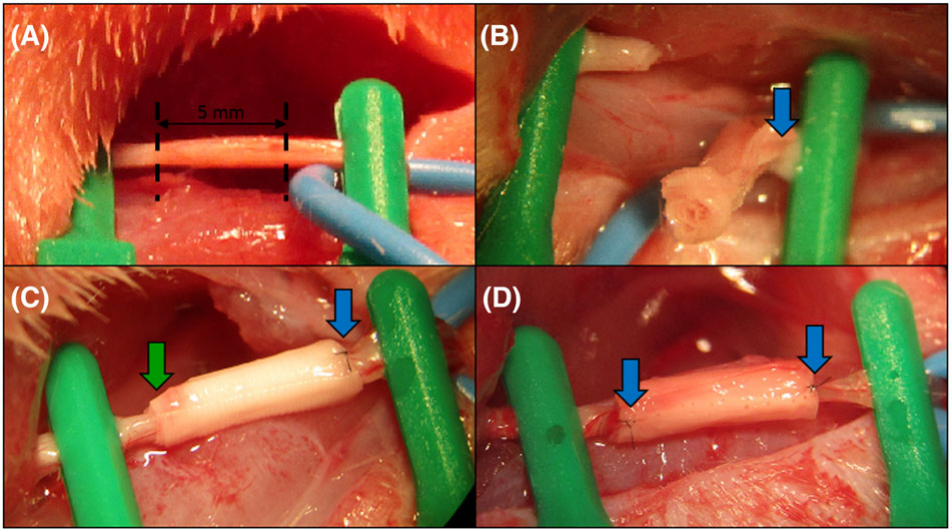
Implantation procedure: A, the rat sciatic nerve was exposed and secured (dashed lines indicate where the nerve was severed with surgical scissors; the portion of nerve between dashed lines was extracted); B, the implant was subsequently sutured once at 0° and once at 180° at the distal nerve segment; C, the proximal nerve segment was placed inside the implant cavity; D, the implant was sutured once at 0° and once at 180° at the proximal nerve segment. Blue arrows indicate the point at which the device was sutured to the nerve epineurium. The green arrow highlights the secured nerve before suture to the device

**Figure 9:**
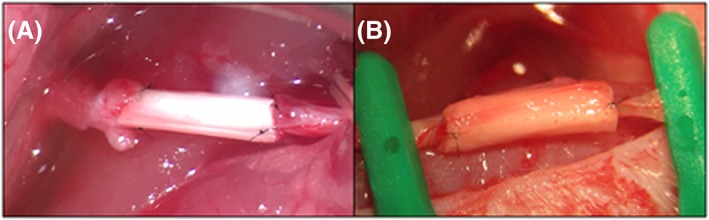
A, Aligned, multi‐channeled silk fibroin nerve guidance conduit sutured between two sciatic nerve segments. B, Tri‐layered, multi‐channeled silk fibroin nerve guidance conduit with jacket layer sutured between two sciatic nerve segments

As a preliminary test to simulate the procedure carried out by a surgeon when performing a nerve repair intervention and to evaluate the surgical handling of the guide, surgery was carried out on the right sciatic nerve of 12 male Sprague Dawley rats. Implantation of the tri‐layered jacketed nerve guidance conduit is depicted in Figure 8. The implant was sutured to the distal nerve segment while avoiding any disturbance to the nerve fascicles due to the small pocket provided by the jacket layer enveloping the multi‐channeled conduit. The implant was then readily positioned to be sutured to the proximal nerve segment as the hollow pocket secured the nerve in the correct position for the final sutures, as demonstrated in Figures 8C and 9B.

The aligned nerve guidance conduit without a jacket layer was sutured between severed segments of the sciatic nerve and is pictured in Figure 9A. Due to the blunt edge of this implant, the nerve fascicles had limited space during the epineurial suture. In consequence, the nerve segment edges were slightly deformed while performing the micro‐suture.

### 3.5 In vivo study

A direct end‐to‐end suture is the preferred method for repairing nerves. Only when the gap between nerve segments is too large and end‐to‐end suture will put excessive tensile stress on the nerve would a graft be a more suitable repair technique.^8^ Sciatic nerve sections from an area distal to the site of injury were taken after 4 months or 8 months in vivo from nerves repaired with either a direct suture or a silk fibroin‐based nerve guidance conduit. All nerve sections were taken within 10 mm distal to the most distal suture at the site of injury or from an uninjured sciatic nerve.

**Figure 10:**
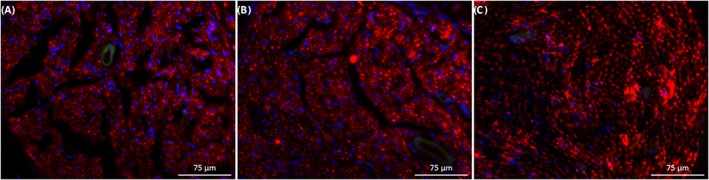
Immunostained nerve sections visualized with fluorescence microscopy at 20x magnification. A, Nerve section taken within 10 mm distal to the silk fibroin‐based implant 4 months after surgery. B, Nerve section taken within 10 mm distal to a direct nerve suture 4 months after surgery. C, Nerve section taken from uninjured rat sciatic nerve. Nerve fibers are represented in red (anti‐β‐tubulin III), and cell nuclei are represented in blue (DAPI). Images representative of triplicate tests performed independently.

Samples taken from experimental groups after 4 months in vivo, plus a control sample taken from an uninjured sciatic nerve, were immunostained for visualization of regenerated nerve fibers (Figure 10). In all samples, abundant neuron regeneration was seen in direct suture nerves as well as in nerves having received a nerve guidance conduit. In addition, samples from both experimental groups showed a homogenous distribution of nerve fibers throughout the nerve fascicles similar to the control sample.

**Figure 11:**
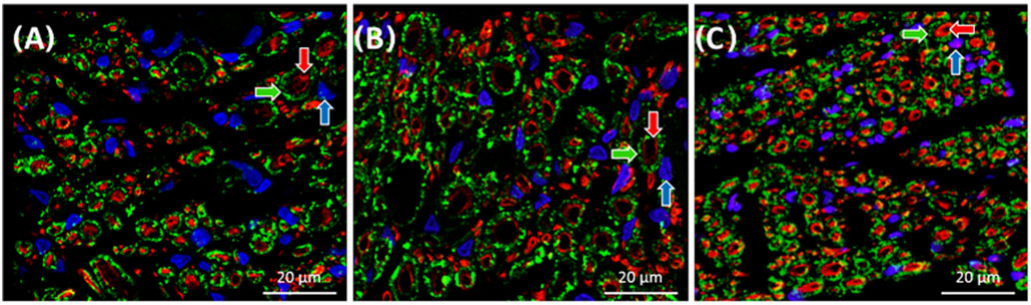
Immunostained nerve sections visualized with a confocal microscope at 40× magnification. A, Nerve section taken distal to the silk fibroin‐based implant 8 months after surgery. B, Nerve section taken distal to a direct nerve suture 8 months after surgery. C, Nerve section taken from uninjured rat sciatic nerve. Nerve fibers are represented in red (anti‐β‐tubulin III), myelin sheaths produced by Schwann cells are represented in green (anti‐myelin protein zero), and cell nuclei are represented in blue (DAPI). Blue arrow indicates Schwann cell nucleus, green arrow indicates myelin sheath, and red arrow indicates enveloped nerve fiber

After 8 months in vivo, both experimental groups showed improvement in nerve regeneration compared with the results obtained at 4 months, as seen in Figure 11. First, visualization of microtubules stained by anti‐β‐tubulin III antibody in regenerated nerve fibers allowed for the measurement of their diameters. At 4 months, nerve fiber diameters in both experimental group samples were too small to be accurately measured. At 8 months, 50 randomly chosen axons' diameters were measured in each sample. Average diameters of axons in the direct suture experimental group sample and the silk fibroin‐based implant experimental group sample were found to be 2.8 ± 1.0 μm and 2.4 ± 0.7 μm, respectively. The average axon diameter from the control group sample was found to be 2.6 ± 1.0 μm. As there were both small‐diameter and large‐diameter axons found in the sciatic nerve, a wide range of axon diameters was to be expected in each sample.

In addition to regenerated nerve fibers, myelin sheaths could also be observed enveloping the large‐diameter nerve fibers in both experimental group samples (Figure 11). Myelin sheath thickness was measured in all samples, and the average myelin thicknesses in the direct suture group sample, the silk fibroin‐based implant group sample, and the control sample were found to be 1.2 ± 0.3, 0.7 ± 0.3, and 1.2 ± 0.2 μm, respectively. In all samples, small‐diameter axons could also be observed without myelin sheaths. These axons were not enveloped in a myelin sheath likely because their diameters were inferior to the critical diameter, 0.7 μm, for activating Schwann cell differentiation to a myelinating Schwann cell.^21^

## 4 DISCUSSION

For over 30 years, the nerve autograft has been considered the gold standard for nerve repair, yet it has been reported to only result in successful functional recovery in 50% of patients.^10^ A significant structural advantage of the autograft, however, is the presence of pre‐established pathways through which axons are guided until they reach the distal section of the severed nerve.^15^ The hollow nerve guides currently found on the market, such as Neuroflex or NeuraGen, lack this important factor that ensures that axons do not become misdirected in pursuit of successful reinnervation with their innervation targets. This guidance component is not lacking, however, in the herein presented implant design, due to the aligned silk nanofiber surface of the micro‐channels. By creating a multi‐channeled tube, the interior surface area of the material is significantly increased, thus improving the guidance capabilities of the conduit.

Contrary to a hollow nerve guide, a multi‐channeled conduit presents an immediate concern resulting in surgical complications that is constantly overlooked in the literature (eg, Yao et al, Bender et al, Dinis et al).^17^, ^22^, ^23^ A hollow nerve guide is easily sutured to the epineurium of each nerve segment without disturbing the positions of the fascicles, both at the proximal and distal nerve segments. A multi‐channeled conduit, however, has blunt edges and risks the deformation of nerve fascicles, as the outer layer of the conduit is sutured directly to the edge of the nerve segments' epineurium. After the nerve is sectioned, the epineurium retracts leaving protruding nerve fascicles, making fascicle deformation virtually impossible to avoid. Responding to this concern, the presented implant design features a “jacket” layer enveloping the guidance conduit. The jacket, which is 1 mm longer than the multi‐channeled conduit on each end, presents a hollow cavity for the placement of each nerve segment before it is sutured to the epineurium. This allows the proximal and distal nerve fascicles to be placed in contact with the multi‐channeled conduit without deforming the natural paths of the regenerating axons.

The presented tri‐layer material used to manufacture the nerve guidance conduit optimizes both surface architecture and mechanical properties in order to incorporate all necessary characteristics of an effective nerve guide, while also taking into consideration the complications of the surgical procedure. First, the tri‐layer material exhibits two surface layers of aligned silk fibroin fibers, with an average diameter of 417 nm ± 134 nm. The aligned fiber surface of the material is a crucial property allowing maximal guidance of the regenerating neurons, which is supported both by Dinis et al, who have shown the capability of neurites to attach and grow along silk nanofibers, and Qu et al, who have demonstrated that smaller fiber diameters (eg, 400 nm) result in more efficient neurite attachment and extension, compared with larger fiber diameters (eg, 1.2 μm).^14^, ^24^ Furthermore, Madduri et al have demonstrated significantly increased neurite outgrowth of sensory neurons and glial cell migration on aligned silk fibroin fiber surfaces compared with randomly deposited fiber surfaces.^25^

Structure and functionalization are frequently the primary focus of a tissue engineered nerve guidance conduit, but analysis of the implantability of the guidance conduit is a fundamental exercise regularly overlooked in the literature. Yet, if the implantation procedure of a nerve guide is overly complex, mistakes could result in reduced effectiveness or the surgeon may simply prefer to use a more reliable guide. The randomly deposited center fiber layer, therefore, proves to be an important component that improves the mechanical properties of the material in order to enhance the ease of implantation.

Clear mechanical benefits of the tri‐layered material, with respect to the purely aligned material revealed by the tensile strength test results, were the comparable stiffness, or resistance to deformation, with an increase in the ductility of the material. An increased material stiffness, represented by a high Young's modulus, results in an implant more resistant to permanent deformation when enduring the various loads involved, for instance, with a person's everyday movements. The significant increase in ductility of the tri‐layer material is represented by the necking observed from the tensile strength tests and the continuity of the tri‐layer material that was kept long after reaching ultimate strength. In contrast, the aligned fiber material met complete or quasi‐complete rupture immediately after ultimate strength was reached. The increased ductility, together with a comparable resistance to deformation, is a benefit for a material used to create a nerve guide in order to ensure that the implant will not fail if the implant experiences an exceptionally large load. Despite the slight decrease in tensile strength with the addition of the center random fiber layer, the tri‐layer material has a greater capability than the aligned fiber material to keep continuity after experiencing a similar load that reaches or exceeds slightly the maximum tensile stress of either material.

An additional mechanical property that needs to be addressed for a nerve guide is the material's resistance to tearing. Nerve guides are often sutured to the epineurium during the surgical procedure, which requires a puncture to the material very close to the material edge.^15^ If the material has a poor resistance to tearing, a small load from the suture thread after puncturing the material will cause the implant to detach from either nerve segment, and the implant will fail. The tri‐layer material was shown to possess a much higher resistance to tearing than the aligned material. With the addition of the center random fiber layer, the maximum force withstood from the sutured tri‐layer material increased by 371.6%. In addition, the elongation of the sutured tri‐layer material at its maximum force was 734.5% higher than that of the aligned material. There are two main benefits of this significant increase of the maximum force and elongation withstood by the tri‐layer material. The first, is an increased resistance to tearing, which will more successfully prevent the implant from detaching from the nerve segments in the weeks following the surgery. The second benefit is the increased ease for surgeons to successfully suture the device while devoting less attention to the force being applied to the device during suture.

This novel implant's design application efficacy was compared with a blunt‐edged multi‐channel design using purely aligned silk fiber material. All surgeons participating in this work highly preferred the presented system for three main reasons. The first benefit observed was the ease of placement of the implant for suture. Unlike with the blunt‐edged implants, the jacketed design allows a hollow cavity for each severed nerve edge and does not distort the protruding nerve fascicles. The second advantage of the jacketed implant was the ability of the same cavity to gently secure the nerve in place before applying the sutures. This allowed the surgeon to carefully adjust the position of the nerve to more easily ensure that the nerve segments were not twisted, as twisted nerve segments can significantly hinder successful functional recovery. Finally, it was confirmed that the tri‐layered material was much better adapted for micro‐suture than the purely aligned material, as it successfully resisted tearing.

While attempting to suture the aligned material to the nerve segments' epineurium, the surgeon encountered a recurring complication that did not exist with the tri‐layered material. While attempting suture, the material often teared as a consequence of the trauma from the puncture. This was due (1) to the poor tear strength of the material and (2) to the necessary placement of the suture. Since the multi‐channeled conduit made from the aligned material contained blunt ends, the surgeon was forced to pierce the material for suture very close to the edge of the material itself, in an attempt to avoid deforming the nerve fascicles as much as possible. The consequences of this inconvenience were first, that the surgeon was obligated to use a smaller needle (10‐0 round bodied) than was used for the tri‐layered material (9‐0 round bodied). The need for a smaller needle, consequently, requires a higher level of skill and concentration from the surgeon. Second, the surgeon was sometimes required to make multiple attempts to achieve a clean suture of the material. This resulted in a loss of time and the need for greater precision and more delicate gestures. Third, the surgeon was only satisfied with the stability and positioning of the connection after administering four micro‐sutures for each nerve segment (at 0°, 90°, 180°, 270°). In contrast, the surgeon reported a superior connection using the jacketed device for which the nerve fascicles were gently brought in contact with the edge of the micro‐channeled conduit, only requiring two sutures (at 0° and 180°).

Due to the adapted implant design of the jacketed guidance conduit, the surgeons also suggested that it may be possible to use fibrin glue instead of traditional sutures, which has demonstrated advantages in some cases compared with traditional sutures, such as reducing operative time and suture‐associated inflammation.^26^ This could, therefore, serve as an alternative coaptation method for all surgeons performing the implantation, and would provide surgeons with the convenience to choose their personally preferred method for each unique case.

Once implanted, it is important that Schwann cells can migrate through the guidance conduit and reach the regenerating nerve fibers. After Wallerian degeneration takes place, Schwann cells naturally proliferate and form Bands of Büngner that help guide regenerating axons towards their respective innervation targets, in part by presenting signaling proteins on their surface that attract nerve fiber extension.^5^ The implant material was, therefore, tested in vitro in order to evaluate the behavior of Schwann cells on the material surface. Schwann cells were found to respond positively to the aligned fiber surface of the material by aligning themselves in the direction of the fiber alignment and interacting mainly with cells on the length of the same fiber. This is compared with Schwann cells cultivated on a glass coverslip that display no apparent alignment. The alignment of Schwann cells is critical to guiding the nerve fibers towards their respective innervation targets, and the aligned fiber surface of the implant material has shown to support continued alignment of Schwann cells that may assist in the extension of Bands of Büngner through the guidance conduit. Contrarily, random positioning of Schwann cells throughout the guidance conduit could cause the nerve fibers to take different paths leading to incorrect tissues.

In order to evaluate the success of nerve regeneration using the presented device, an in vivo study using Sprague Dawley rats was performed. Nerve regeneration was evaluated at 4 months and 8 months after injury to the sciatic nerve. When possible, end‐to‐end nerve sutures are the preferred method of surgeons^8^; therefore, the device developed in this study has been compared with the end‐to‐end nerve suture method. At 4 months after injury, histological analysis revealed that there was already successful regeneration of nerve fibers in both the implant and the direct suture experimental groups. Immunostained cross sections of the injured nerve showed a good distribution of nerve fibers from both experimental groups compared with the healthy tissue, meaning that axons throughout the entire nerve were able to successfully grow through the implant and find paths at the distal portion of the severed nerve. Although comparable in abundance, nerve fibers in the nerve guidance conduit sample appeared to have slightly smaller diameters than those found in the direct suture sample. Smaller diameters could signify slightly slower regeneration, as Ikeda and Oka demonstrated that average axon diameter increases over time during nerve regeneration.^27^ This minor hindrance in axon growth may be due to a combination of factors, including the additional time for proliferating Schwann cells to migrate through the length of the implant, the delay of the growth cones on the ends of axons to encounter bio‐signaling cues from proliferating Schwann cells, and the extra distance needed to be traveled by the nerve fibers through the guidance conduit. Axons grow at a speed of about 1 mm per day,^28^ and, therefore the addition of a 5‐mm guidance conduit would set regeneration time behind several days compared with an end‐to‐end suture method.

Immunostained cross sections at 8 months after injury first showed larger axon diameters in both experimental groups compared with samples taken at 4 months. While nerve guidance conduit group axons were confirmed to have a smaller diameter compared with direct suture group axons, standard deviations for each group, including the control group, were very high, which is normal, as the sciatic nerve naturally contains both small‐diameter and large‐diameter axons. The average axon diameters on both experimental groups at 8 months were, therefore, comparable to each other and to that of the control group. Similarly, myelin sheath thickness was found to be comparable between all group samples, with a slightly lower average found in the nerve guidance conduit group. While there are several factors that determine conduction velocity, myelin sheaths play an important role.^29^ Therefore, the regeneration of myelin around large‐diameter axons is a distinct signifier of successful regeneration of efficient communication between peripheral tissues and the CNS in both experimental group samples.

From this long‐term evaluation of nerve regeneration, results from the implantation of the nerve guidance conduit developed in this study have proved comparable to the preferred method of nerve repair by surgeons. When the gap between severed nerve segments is too large, the direct suture is not possible, and a different method must be used to connect the proximal and distal nerve portions. In this study, we have demonstrated that this nerve guidance conduit is a good alternative nerve repair method, which can promote satisfactory regeneration of nerve fibers along with the renewal of myelin sheaths around large‐diameter axons.

This study emphasizes the importance of the design of a nerve guidance conduit including the choice of biomaterial used, the material fabrication technique and organization, and the architecture of the three‐dimensional device itself. This foundational design for a nerve guidance conduit has shown to promote nerve regeneration success comparable to that of an end‐to‐end suture at the rat sciatic nerve long term. Histological results revealed that the main difference between this nerve guidance conduit and the end‐to‐end suture interventions was a slightly delayed restoration of nerve fiber diameter size and myelin sheath thickness, signifying a slightly delayed regeneration. While possible explanations for these results have previously been expressed, encouraging a more accelerated regeneration time may be explored by expanding upon the nerve guidance conduit model presented in this study. For example, the functionalization of the material with growth factors may promote accelerated nerve fiber extension. Growth factors that have already been explored to promote enhanced nerve regeneration include nerve growth factor, ciliary neurotrophic factor, neurotrophin‐3, and glial derived neurotrophic factor.^5^ For example, Wang et al found more regenerated fibers and a larger gastrocnemius muscle weight ratio from poly (DL‐lactide‐co‐glycolide) nanofiber nerve guidance conduits functionalized with nerve growth factor compared with non‐functionalized poly (DL‐lactide‐co‐glycolide) conduits.^30^ Furthermore, Zhang et al showed that injecting ciliary neurotrophic factor within a silicon guidance conduit increased the diameter and degree of myelination of regenerated axons compared with empty guidance conduits.^31^ In addition, the seeding of Schnwann cells within the device may enhance nerve regeneration efficacy. For example, Ladak et al demonstrated an increase in regenerated motor neurons from collagen nerve guidance conduits seeded with Schwann cell‐differentiated mesenchymal stem cells compared with empty conduits in the rat.^32^ Therefore, this study presents a strong foundation nerve guidance conduit design that may be investigated further by various functionalization techniques in order to develop a preferred alternative to the autograft.

The implants in this study were sterilized using 70% ethanol solution which is an effective disinfectant for most bacteria and virus; however, more thorough methods of sterilization would need to be implemented for commercial development of silk fibroin fiber‐based material systems. Additional studies should be conducted to evaluate the structural stability of the silk fibroin nanofiber material under other sterilization techniques such as autoclaving and ultraviolet radiation.

## 5 CONCLUSION

The jacketed, micro‐channeled conduit based on a nanofibrous material organized in a tri‐layered architecture presented in this work is a base design for a nerve guidance conduit that addresses the surgical application concerns that are regularly overlooked in the literature. As a result of the micro‐channels, the device exhibits a high surface area to volume ratio providing an aligned nanofiber surface in order to increase axon guidance during regeneration. Furthermore, the conduit contains a tri‐layered jacket, which significantly increases the ease of surgical implantation. Histology analyses throughout a long‐term in vivo study showed that nerve regeneration using this nerve guidance conduit was comparable to regeneration results from an end‐to‐end suture, which is the preferred technique of nerve repair if the gap between severed nerve segments is minimal. In addition, the device may be adapted further to ameliorate nerve regrowth efficacy by modifications of the material or bio‐functionalization. Silk fibroin was used for this model because of the advantageous properties for biological devices. Silk fibroin is a natural, biocompatible polymer with robust mechanical properties that is biodegradable, easily chemically modifiable, and easily functionalized. Therefore, silk fibroin supports the incorporation of many functionalizing materials or bio‐signaling molecules in order to improve the performance of the device.

## CONFLICT OF INTEREST

The authors have declared that there is no conflict of interest.

## AUTHOR CONTRIBUTIONS

Conceptualization: Kayla Belanger, Guy Schlatter, Anne Hébraud, Frédéric Marin, Bernard Devauchelle, Christophe Egles

Funding Acquisition: Christophe Egles, Bernard Devauchelle

Investigation: Kayla Belanger, Bernard Devauchelle, Sylvie Testelin, Stéphanie Dakpé

Writing—original draft: Kayla Belanger, Christophe Egles, Guy Schlatter

Writing—review and editing: Kayla Belanger, Christophe Egles, Guy Schlatter

## Supporting information


**Figure S1**. The fabrication method of a jacketed, multi‐channeled nerve guide based on the tri‐layered electrospun silk fibroin nanofiber material is shown. (A) A 5 mm by 3 cm tri‐layered nanofiber material is first rolled around a Teflon‐coated stick (0.2 mm in diameter) perpendicular to the surface fiber alignment. After a full rotation, a second Teflon‐coated stick is added and the material is rolled an additional full rotation. (B) This process is continued until the material is rolled completely allowing the incorporation of between 13 and 18 Teflon‐coated sticks of equal diameter. The rolled material is immersed in methanol to induce β‐sheet formation. The material is dried at room temperature for 1 hour and the Teflon‐coated sticks are easily removed creating a multi‐channeled tube. (C) The multi‐channeled tube is placed at the edge of a 7 mm by 3 cm tri‐layered nanofiber material. (D) The material is rolled perpendicular to the surface fiber alignment around the multi‐channeled tube for 3 full rotations to create a jacket layer. The jacketed, multi‐channeled material is then water vapor annealed at room temperature for 4 hours to induce β‐sheet formation.
