# Supplementary material for: A multi‐layered nerve guidance conduit design adapted to facilitate surgical implantation
Source: Health Sci Rep. 2018 Oct 31;1(12):e86. doi: 10.1002/hsr2.86 (PMC6295612; doi:10.1002/hsr2.86)
Supplement: Supplementary file 1 — Figure S1. The fabrication method of a jacketed, multi‐channeled nerve guide based on the tri‐layered electrospun silk fibroin nanofiber material is shown. (A) A 5 mm by 3 cm tri‐layered nanofiber material is first rolled around a Teflon‐coated stick (0.2 mm in diameter) perpendicular to the surface fiber alignment. After a full rotation, a second Teflon‐coated stick is added and the material is rolled an additional full rotation. (B) This process is continued until the material is rolled completely allowing the incorporation of between 13 and 18 Teflon‐coated sticks of equal diameter. The rolled material is immersed in methanol to induce β‐sheet formation. The material is dried at room temperature for 1 hour and the Teflon‐coated sticks are easily removed creating a multi‐channeled tube. (C) The multi‐channeled tube is placed at the edge of a 7 mm by 3 cm tri‐layered nanofiber material. (D) The material is rolled perpendicular to the surface fiber alignment around the multi‐channeled tube for 3 full rotations to create a jacket layer. The jacketed, multi‐channeled material is then water vapor annealed at room temperature for 4 hours to induce β‐sheet formation. [file HSR2-1-e86-s001.zip › Supplementary Material_Figure S1.docx]

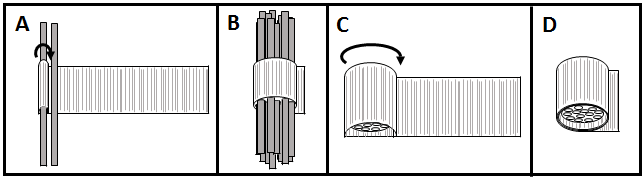


**Figure S1.** The fabrication method of a jacketed, multi-channeled nerve guide based on the tri-layered electrospun silk fibroin nanofiber material is shown. (A) A 5 mm by 3 cm tri-layered nanofiber material is first rolled around a Teflon-coated stick (0.2 mm in diameter) perpendicular to the surface fiber alignment. After a full rotation, a second Teflon-coated stick is added and the material is rolled an additional full rotation. (B) This process is continued until the material is rolled completely allowing the incorporation of between 13 and 18 Teflon-coated sticks of equal diameter. The rolled material is immersed in methanol to induce β-sheet formation. The material is dried at room temperature for 1 hour and the Teflon-coated sticks are easily removed creating a multi-channeled tube. (C) The multi-channeled tube is placed at the edge of a 7 mm by 3 cm tri-layered nanofiber material. (D) The material is rolled perpendicular to the surface fiber alignment around the multi-channeled tube for 3 full rotations to create a jacket layer. The jacketed, multi-channeled material is then water vapor annealed at room temperature for 4 hours to induce β-sheet formation.
